# Foliar Application of a Biostimulant Based on Fermented Pomegranate Waste Increases Tomato Yield in Greenhouse

**DOI:** 10.1155/sci5/9995637

**Published:** 2026-01-09

**Authors:** Gilberto Abdón-Aguilar, Ana L. Rueda-Altunar, Armando Robledo-Olivo, Susana González-Morales, Adalberto Benavides-Mendoza, Ana Verónica Charles-Rodríguez, Antonio Juárez-Maldonado, Marcelino Cabrera-De la Fuente

**Affiliations:** ^1^ Horticulture Postgraduate Program, Food Science and Technology Department, Universidad Autónoma Agraria Antonio Narro, Saltillo, Coahuila, Mexico, uanl.mx; ^2^ Fermentations and Biomolecules Lab, Food Science and Technology Department, Universidad Autónoma Agraria Antonio Narro, Saltillo, Coahuila, Mexico, uanl.mx; ^3^ SECIHTI-UAAAN, Horticulture Department, Universidad Autónoma Agraria Antonio Narro, Saltillo, Coahuila, Mexico, uanl.mx; ^4^ Horticulture Department, Universidad Autónoma Agraria Antonio Narro, Saltillo, Coahuila, Mexico, uanl.mx; ^5^ Department of Botanic, Universidad Autónoma Agraria Antonio Narro, Saltillo, Coahuila, Mexico, uanl.mx

**Keywords:** *aspergillus*, liquid fermentation, nutrients, phenolic compounds, plant biostimulant, tomato

## Abstract

The use of biostimulants can help to mitigate the conditions of biotic and abiotic stresses in crops by enhancing the crop yield and product nutrients. The novelty of this research was to produce a biostimulant for the tomato cultivation through fermentation of pomegranate waste, evaluating in the crop the effect on the growth, development, and quality of tomato fruits. Pomegranate bagasse was used as a substrate during the liquid fermentation using *Aspergillus niger* M4 strain. Three applications of fermented extract were made in three phenological crop stages for each of the four different treatments. The biotechnological process allowed the transformation of pomegranate residues, increasing the content of antioxidant activity and catechin in 467% and 315%, respectively. The fermentation enabled the mineral content modification, such as the condensed tannins, zinc, magnesium, and antioxidant capacity. By applying the fermented extract of pomegranate, an increase of 34% in crop yield and a 32% in the lycopene content in tomato fruit was obtained. The use of a fermentative process enables the pomegranate waste mineral modification, enhancing the biostimulant capacity of pomegranate residues. The foliar application of a raw pomegranate fermented extract increases the crop yield and nutritional quality of the tomato fruits.

**Statement of Novelty**

• Bioprocess of the waste derived by a pomegranate juice company to change its composition and use it as a biostimulant to increase the yield and nutritional composition in tomato plants.

## 1. Introduction

Overpopulation requires maximizing agricultural production, with crops that can withstand the environmental inclemency generated by global warming, fulfilling the nutritional demand. The crop development can be affected by several factors, which generate stress in the plant and that can be from an abiotic or biotic source [1]. Stress conditions can be supported and, in some cases, be reversed by plants through the generation of systemic defense mechanisms, which include the generation of secondary metabolites with the involvement of the primary carbon metabolism [2, 3]. Those metabolites are mainly integrated by phenolic compounds (PhC), which conforms the antioxidant defense system, being the main mechanism for the elimination of reactive oxygen species and the first line of defense of plants [4]. PhC are synthesized by the shikimate/phenylpropanoid or acetate‐malonate/polyketide metabolic pathways. The glycolytic and pentose phosphate pathways provide precursors (phosphoenolpyruvate and erythrose‐4‐phosphate, respectively) to the shikimate pathway to produce phenylalanine and tyrosine as the precursor of phenylpropanoid metabolism that, in turn, feeds the various specific flavonoid pathways [5].

PhC constitute a wide group of chemical substances, such as tannins, lignans, stilbenes, coumarins, quinones, xanthones, phenolic acids, flavones, flavonols, catechins, anthocyanins, and proanthocyanins, which due to their redox properties can act as donors of hydrogens, to prevent or delay the development of degenerative diseases [5, 6]. Defense mechanism processes can increase the demands for energy, decreasing the development and growth of the plant, as well as a low quantity and quality of fruits [7]. Carbon limitation triggers a dramatic reprogramming of metabolism and gene expression, limiting growth and ensuring energy homeostasis and survival, minimizing the nutritional content in fruits [8, 9].

Biostimulants are compounds that have active principles, which act on the physiology of plants, increasing their growth, productivity, and the quality of the fruit [10]. In recent years, biostimulants have been developed in the agricultural market, which are used to enhance size, color, and shape, in addition to rise crop yield, activating the development of different organs and reducing the damage caused by biotic and abiotic stresses [11]. PhC can not only function as biostimulants for yield improvement but also have the ability to function as antimicrobial agents, both in vitro and directly on plants. For example, Buchmann et al. [12] reported in vitro inhibition of seven phenolic phytochemicals (gallic acid; epicatechin; epigallocatechin gallate; daidzein; genistein; myricetin; 3‐hydroxy‐6‐methoxy flavone) in combination with six antibiotics against *Acinetobacter baumannii, Escherichia coli, Klebsiella pneumoniae, Pseudomonas aeruginosa,* and *Staphylococcus aureus.* Also, Abdelkhalek et al. [13] proved a decrease in the incidence of *Fusarium culmorum*, *Rhizoctonia solani,* and tobacco mosaic virus (TMV) in the plant of *Chenopodium amaranticolor* when applied in a sprayed way 2% polyphenolic extracts of resveratrol, kaempferol, myricetin, rutin, quercetin, and rosmarinic acid. From this outlook, the foliar application of a raw extract having PhC obtained from a fermentation process will increase the productivity and nutritional quality of the fruits [14]. The bioprocessing of phenolic‐rich agrowastes can lead to an organic extract, which acts as a plant biostimulant giving a value added for the waste from the pomegranate juice industry [15].

Evidence shows that the shell of the pomegranate is an excellent substrate for the microbial production of secondary metabolites of the highest antioxidant capacities, such as hydrolyzable ellagitannins (ellagic acid, punicalagin, punicalin, and gallic acid) [16]. The highest concentration of these compounds is found in the peel (exocarp and mesocarp), which forms about 50% of the whole fresh fruit [17]. In México, more than 10,090 tons of pomegranate cultivation were generated during 2022 in 18 states of national territory [18]. Globally, around 3.8 million tons are produced annually [19] consumed as fresh juice, canned beverages, jelly, jam, and paste for flavoring and coloring drinks [20]. Several reports show the enrichment of the antioxidant potential of fermented substrates and the influence of different physicochemical parameters of fermentation [21]. However, no studies were found that find the mineral modification of the pomegranate fermented extract, and the evaluation of its application on the tomato crop to increase yield and fruit quality. The application of fermented pomegranate extracts (FPEs) could be a novel application for agricultural processes, giving an added value to the pomegranate juice industry and the valorization of its waste. In this matter, pomegranate peel being a rich source of PhC has demonstrated biostimulant effects for crops and biofertilizer effect when applied in soil. For example, Bodor et al. [22] when applied to PhC reported a biostimulant effect on oilseed rape (*Brassica* napus), improving germination indices and promoting the development of aerial plant parts. Therefore, this paper attempts to find the changes involved in the biodegradation of pomegranate residues, which allow the development of a fermentation extract with the possibility of function as a biostimulant in agricultural crops.

## 2. Materials and Methods

### 2.1. Microorganism and Raw Material

The fungus *Aspergillus niger* M4 was obtained from the collection of the Fermentation and Biomolecules Lab at the Department of Food Science and Technology of the Universidad Autónoma Agraria Antonio Narro (UAAAN) (access code KY825168.1). The strain was propagated on potato dextrose agar (PDA) (BD Bioxon, México) at 35°C for a period of 5 days. Reserve cultures were kept in PDA at 4°C and were cultured routinely.

Pomegranate (Wonderful variety) waste (PGW) is derived from the process of obtaining pomegranate arils by hand from the company “El Baluarte,” found in the municipality of Cuatro‐Ciénegas, Coahuila, Mexico. The PGW consisted mainly of husk (exocarp) and membrane (mesocarp) material, obtained from the manual separation from pomegranate arils (seeds). PGW was obtained during September 2017. The obtained PGW was dehydrated in a convection oven (Biobase Biodustry BOV‐T70C) at 70°C for 24 h. Subsequently, the samples were shredded and sequentially sieved (Montinox, México) in particle diameters in the range of 0.60–0.85, 1.00–1.25, and 2.0–2.5 mm (sieve range 10–30). PGW powder was further used as a substrate for analysis and fermentation process.

### 2.2. Physicochemical Characterization

A proximal analysis was conducted, which consisted of the triplicate determination of total dry matter, humidity, ash, fat, protein, and crude fiber by the procedures described by the AOAC [23]. Total sugars were also determined by the phenol‐sulfuric method proposed by Dubois et al. [24] at 480 nm (UV/VIS Spectrophotometer, VELAB). The reducing sugar content was evaluated using the method reported by Miller [25], using dinitro‐salicylic (DNS) acid reagent (Sigma Aldrich, USA). The total phenol content (TPC) was determined using the Folin Ciocalteu reagent (Sigma Aldrich, USA) at 790 nm, Singleton et al. [26] (UV/VIS Spectrophotometer, VELAB) and reported as milligrams of gallic acid equivalents by grams of dry substrate (mg GAE gds^−1^). The condensed tannin content (CTC) was determined as described by Ventura‐Sobrevilla [27], and the results were expressed as mg of catechin equivalents per gram of dry substrate (mg CE gds^−1^).

### 2.3. Fermentation Process in Liquid Medium

A modified Czapek–Dox culture media was prepared, consisting of (g L^−1^) (all reagents from JT Baker, USA): PGW powder (25) carbon source, urea (8.76) as a nitrogen source, KH_2_PO_4_ (4.38), MgSO_4_•7H_2_O (0.88), CaCl_2_ Anhydrous (0.09), MnCl_2_•4H_2_O (0.02), Na_2_MoO_4_•2H_2_O (0.01), FeSO_4_•7H_2_O (0.12). Erlenmeyer flask of 250 mL was placed with 1.25 g of PGW powder and 50 mL of culture medium in each flask. They were inoculated with a concentration of 1 × 10^6^ spores per mL. The flasks were incubated at 30°C and 175 rpm (New Brunswick™ Innova 44 Incubator Shaker). The fermentation process was monitored 240 h, evaluating every 24 h, taking a flask in each sampling time. The number of total phenols [26], total sugars [24], reducing sugars [25], biomass (gravimetry), and mineral analysis by Inductively Coupled Plasma (ICP, Thermo Jarrel Ash Irish Advantage model 74,400) were determined for each sample. The antioxidant activity was determined by 2,2‐difenil‐1‐picrilhidrasil (DPPH) method [28] with some modifications. The DPPH (Sigma‐Aldrich, U.S.A.) radical was prepared at a concentration of 60 μM in a methanolic solution. For the standard curve, a stock solution at a concentration of 1000 ppm Trolox (Sigma‐Aldrich, U.S.A.) was used, from which different dilutions were prepared. For the reading of the samples, 7 μL of the blank, dilutions of stock solution for the standard curve, and the samples of the different fermentation times (0, 24, 48, 72, 96, 120, 144, 168, 192, 216 and 240 h) were placed on the microplate. Subsequently, 193 μL of the DPPH‐methanol solution were added to each sample. The microplate was covered with aluminum foil to avoid contact with light and allowed to stand at room temperature for 30 min. A Biotek ELx808 microplate reader at an absorbance of 540 nm was used to read the samples. The results are expressed as Trolox equivalent antioxidant capacity per gram of dry substrate (mM TEAC gds^−1^).

### 2.4. Crop Establishment Location

The present investigation was conducted in a greenhouse, located at the Universidad Autónoma Agraria Antonio Narro, in Saltillo, Coahuila, México (25°21′5″ north latitude and 101°1′47″ west longitude, elevation 1742 MASL), in the Horticulture Department. Tomato seed of the Sun 7705 variety (Nunhems) of indeterminate growth was used. The phenotype of the seed is an indeterminate saladette tomato hybrid variety. It is a vigorous plant that produces large and extra‐large fruits (120–160 g) of bright red color. The fruits are uniform, of excellent quality, have the best shelf life, do not present cracking, and have excellent apical closure. A 200‐well polystyrene tray was filled with a mixture of perlite‐peat moss 1:1 (v:v), placing one seed per well. The transplant was performed one month after sowing, for which 10‐L capacity black polyethylene bags were filled with a 1:1 mixture of perlite‐peat moss. Irrigation was applied through a directed irrigation system, using the Steiner [29] nutrient solution for crop nutrition. The greenhouse conditions were an average temperature of 28°C and a relative humidity of 50%–60%.

### 2.5. Application of the Extract to the Crop

The application of the fermented extract of PGW was done via foliar using sprays. The treatments applied were as follows: T0 = modified Czapek–Dox culture media without PGW powder, T1 = 0.75 L ha^−1^, T2 = 1.50 L ha^−1^, T3 = 3.00 L ha^−1^. Three applications were made: the first application was made in the growth and development stage 5 days after transplantation (DAT), the second application (flowering stage) at 20 DAT, and the third application at 35 DAT (filling of fruits).

### 2.6. Evaluation of Agronomic Variables and Fruit Quality

The agronomic variables evaluated were the following: height (cm), stem diameter (mm), number of leaves, fresh and dry aerial biomass (g), fresh and dry root biomass (g) and yield (g). The evaluation of the variables of height, stem diameter, and number of leaves was conducted 24 h after the application of the treatments (first application: growth and development; second application: flowering; third application: filling and fruit set); the absent variables were evaluated at the end of the culture. The crop yield was figured out according to the development of the plant, counting the weight of fruits obtained at the time of physiological maturity, making the final count to the second production cluster (100 DAT). The variables evaluated for fruit quality were firmness, total soluble solids (TSS), titratable acidity, pH, lycopene, antioxidant potential (DPPH), and a mineral analysis. The DPPH scavenging activity was determined with a Trolox (Sigma‐Aldrich, U.S.A.) standard curve, linear between 0.4 and 4.0 mM Trolox L^−1^. The results were expressed as mM Trolox equivalents per gram of dry substrate (mmol TEAC gds^−1^). The collection of 24 fruits was carried out at 60 DAT; these fruits were of uniform size without physical damage, being collected at stage 6 (light red) of maturity according to the USDA visual scale [30]. Fruit firmness was determined with a manual penetrometer (Wagner Instruments model FDK 20, CT, USA). The pH was determined using a HI‐98130 digital potentiometer (Hanna Instruments). TSS were measured by placing a drop of tomato juice on the lens of a digital refractometer (ATAGO, MASTER‐100Hmodel, WA, USA). Titratable acidity was determined using the Official Methods of Analysis of the AOAC [23] colorimetric technique, and the data were expressed as a percentage of citric acid. The variables of lycopene [31], antioxidant potential, and mineral analysis were determined with the techniques previously described.

### 2.7. Experimental Data Analysis

Fermentation experiments were conducted by triplicate, and average values are reported. Data were analyzed using a unidirectional analyses of variance (ANOVA) procedure in Minitab® 17.1.0. When needed, treatment means were compared using Tukey’s range procedure (*p* ≤ 0.05). For the establishment of the culture, a randomized complete block design was used with three treatments and an absolute control with 10 plants per treatment. Statistical analysis was performed using analysis of variance and comparison of means according to Fisher’s LSD (*p* ≤ 0.05), using the InfoStat Software, 2018.

## 3. Results

### 3.1. Proximal Analysis of the Residue

The importance of the proximal analysis (Table 1) of the residue lies in the knowledge of the material as a source of energy and proteins for the proper development of the microorganism and its metabolic processes to conduct the fermentation stages in a stable manner. The results obtained agree with those of other studies [15, 28, 29] except for protein and crude fiber; these are found above. The result of protein is 15.24%; the value obtained for raw fiber is 67.16%. The obtained values of total sugars present in the PGW have a low percentage, while the concentration of reducing sugars was 5.19%. On the other hand, a concentration of total hydrolyzable phenols of 4.19% was obtained. These results show that the concentration of total sugars found in the PGW has a low concentration when compared to that of the whole fruit.

**Table 1:** Physicochemical characterization in dry basis of the PGW and results of its characterization as substrate for the fermentation process.

Parameter	Present work (%)	Robledo et al. [35]	Castillo‐Sánchez [58]	Buenrostro et al. [59]
Total dry matter	92.10 ± 0.014	94.45 ± 1.25	90.75 ± 2.25	NA^∗^
Humidity	7.90 ± 0.014	5.50 ± 1.25	9.40 ± 1.82	11.86 ± 0.05
Carbohydrates	6.63 ± 0.017	NA^∗^	NA^∗^	75.38 ± 0.18
Ash	2.45 ± 0.002	3.59 ± 0.08	3.42 ± 0.97	4.51 ± 0.01
Grease	0.62 ± 0.004	3.57 ± 0.38	3.44 ± 1.09	2.64 ± 0.08
Protein	15.24 ± 0.320	1.26 ± 0.17	4.26 ± 0.23	8.66 ± 0.01
Raw fiber	67.16 ± 0.014	17.75 ± 1.61	14.30 ± 2.61	8.81 ± 0.07
Total sugars (mg mL^−1^)	0.90 ± 0.020	NA^∗^	236.5 ± 0.62	NA^∗^
Reducing sugars (mg mL^−1^)	5.19 ± 0.010	NA^∗^	NA^∗^	NA^∗^
Total phenol content (mg GAE 100 gds^−1^)	14,130 ± 8.15	10,000	8700	NA^∗^

*Note:* Comparisons of the present work and earlier reported works.

^∗^NA: not available.

The protein content gives us a reference to the nitrogen amount, which it is needed by the microorganism to carry out a good development and fermentation process. Our result shows that the powder of PGW is a residue with good characteristics to be used as a substrate in the fermentation in liquid medium, bringing all the necessary nutrients for the microorganism to perform an adequate metabolism. Regarding protein content, Aguilar et al. [32] reports 6.10% of protein after fermentation at 96 h; our result is above the cited author’s result, finding a high protein content. This nutrient is essential to conduct the activity of the microorganism, thus generating more cells and producing compounds. The total fiber present in samples shows a high value in PGW when compared with another works (Table 1). The fiber is the amount of cellulose, hemicellulose, and lignin; these polysaccharides have high molecular weight with phenolic compounds, so the PGW is an excellent source to obtain phenolic substances of high added value. It has been reported that the compounds associated with the fiber of pomegranate husk correspond to hydrolyzable tannins such as punicalagin, punicalin, pedunculagin, among other phenolic compounds such as ellagic acid, gallic acid, rutin, and quercetin [33]. All these compounds can function as a defense system in plant with biotic or abiotic stresses when applied in a foliar method.

### 3.2. Degradation Kinetics of Pomegranate Bagasse

**Figure 1:**
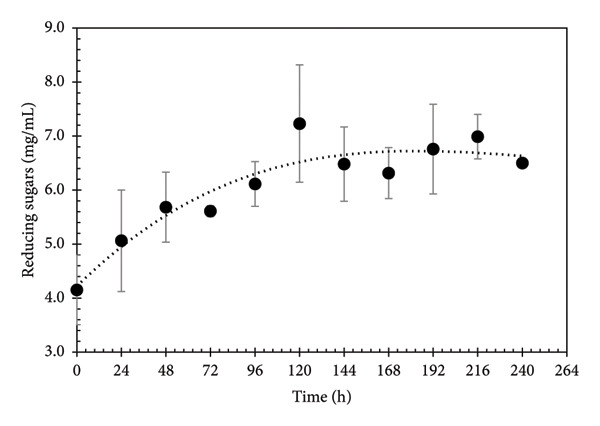
Kinetic monitoring of reducing sugars during the PGW fermentation in liquid media. Dotted line stands for the tendency of values.

The first sources of substrate available in the culture medium are the dissolved sugars, derived from the PGW. The behavior of reducing sugars during fermentative kinetics is shown in Figure 1, where a notable increase is seen at 24 h of fermentation, 120 h being the maximum point of reducing sugar quantification with a value of 7.231 mg mL^−1^, ending with a production of 6.499 mg mL^−1^ at 240 h. Subsequently, a decreasing trend is seen, showing that the PGW has reducing sugars, and that the fungus can metabolize them in lesser amounts. Fadavi et al. [34] mentioned that the first sources of substrate available in a culture medium for microorganisms are the sugars dissolved in it, such as glucose and fructose, considered as easily assimilated sugars. The increase in the sugar content is associated with the metabolic needs of the microorganism, mainly to obtain energy for its growth. The microorganism needs to synthesize enzymes to degrade the polymers to obtain monomeric sugars, which will accumulate in the fermentation broth to be available for the microorganism. The sugar kinetic behavior can be explained since the pomegranate husk not only holds free sugars; it also contains phenolic compounds with small quantities of glucose within, which can be used as a carbon source. There are results that report a content of reducing sugars of 13.02 g mL^−1^ in pomegranate juice for different varieties [33]. Robledo et al. [35] mention that the pomegranate husk is a good support and at the same time an excellent substrate in the production of metabolites of high commercial interest such as ellagic acid due to the degradation of its ellagitannin content.

**Figure 2:**
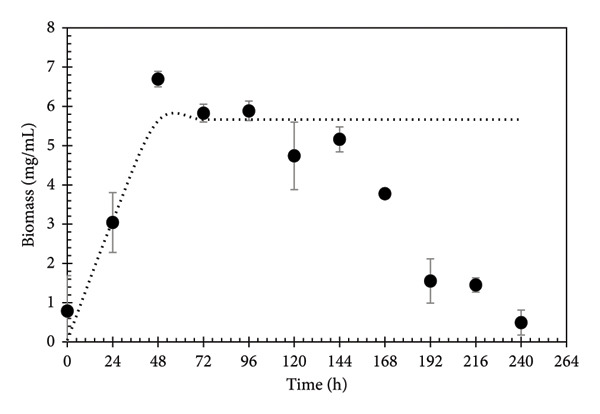
Fungal biomass concentration during the PGW fermentation in liquid media. Dotted line is the Verhulst–Pearl logistic equation adjustment [36].

The biomass production kinetics of *Aspergillus niger* are presented in Figure 2. The graph shows that the growth curve of the microorganism does not have a clear latent phase in the monitored time, while an exponential phase is clearly observed. The moderate growth observed indicates the presence of reducing sugars in the PGW and that the fungus can obtain reducing sugars in small quantities from polymeric fractions present in the fibers of the PGW. The behavior of microbial growth (Figure 2) may be due to the amount of monomeric sugars present, showing a mycelial increase in the microorganism after 48 h, to later show a stationary phase when the nutrients are depleted. In this stationary phase, it is where various enzymes could be synthesized that allow degrading the lignocellulosic material and the different phenolic compounds present.

**Figure 3:**
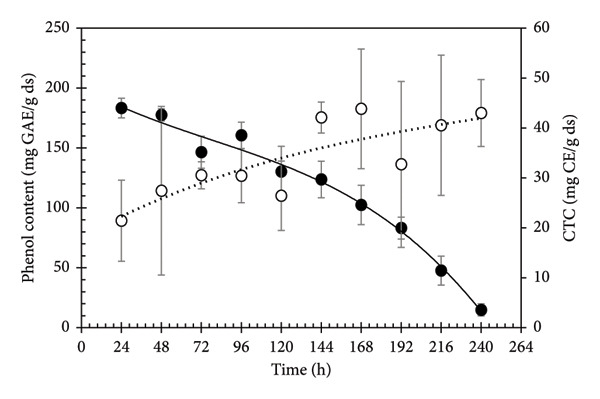
Kinetic monitoring of phenol content (solid circle, straight line) and condensed tannin content (open circles, dotted line) during the PGW fermentation in liquid media. Lines represent the tendency of values.

The consumption of polyphenols and accumulation of condensed tannins are shown as a function of time in Figure 3. From 0 to 48 h, an increase of phenolic compounds is seen, being the maximum in 48 h; afterward, there is a significant decrease due to the constant hydrolysis of polyphenols, observing a significant consumption rate and ending with a value of 150.3 mg mL^−1^ at 240 h. The hydrolyzable polyphenols (HPP) quantified as TPC could be used as a source of carbon and energy by microorganisms; in this case, the strain could grow in high concentrations of these compounds. The content of TPC in the PGW with a final average amount of 14.8 mg GAE gds^−1^ suggests that the synthesis of tannase and the degradation of HPP is linked to the growth of the microorganism, since the degradation of TPC occurs at the points of greatest microbial growth. Payán [37] mentions that by using tannase as a hydrolyzer, 100% of high‐molecular‐weight tannins can be degraded, turning them into lighter compounds such as glucose and gallic acid. Prigione et al. [38] mention that the presence of hydrolyzable tannins (punicalin, punicalagin, and pedunculagin) and flavonoids (catechin and epicatechin) can be used as a carbon source due to the production of the enzyme tannase. This establishes that the microorganism can degrade the polyphenols present in the liquid media. Gutierrez Pacheco [39] and Singh and Immanuel [40] report TPC of 255.98 and 249.41 mg GAE g^−1^, respectively. As seen, the polyphenol content in the current work is similar to other authors. Ozgen et al. [41] reported a range of polyphenol concentrations of 124.5–207.6 mg GAE mL^−1^ for fresh pomegranate aryls juice.

The consumption of HPP is possible since polyphenols of higher molecular weight are degraded to smaller molecules (monomer or dimer). The HPP of pomegranate husk reported in the literature are the ellagitannins [36], such as punicalagin, punicalin, and ellagic acid, which have high antioxidant capacity.

**Figure 4:**
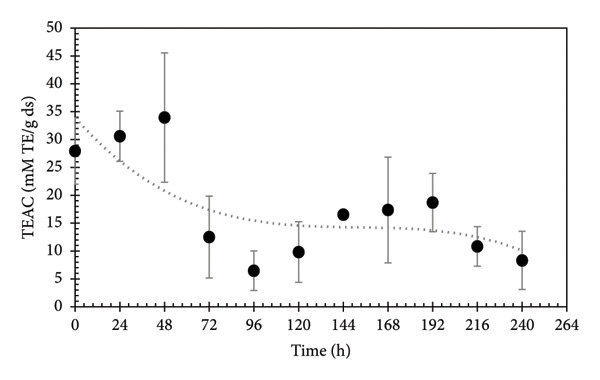
Kinetic monitoring of antioxidant activity as trolox equivalent antioxidant capacity (TEAC) during the PGW fermentation in liquid media. Dotted line represents the tendency of values.

The kinetics of the antioxidant activity from the liquid state fermentation is shown in Figure 4. In the first 48 h, a low percentage of antioxidant activity is observed due to the small number of biomolecules with antiradical capacity. After 72 h, a considerable increase in the antioxidant activity is achieved until 240 h. The production of antioxidant compounds derived from hydrolyzable phenols presents two minimal points, during kinetic fermentation process, at 96 and 240 h. The decrease in gallic acid content (Figure 3) suggests the presence of gallate decarboxylase enzymes, which are responsible for catalyzing the decarboxylation of gallic acid in pyrogallol [38]. Several compounds derived from the HPP degradation are responsible for the antioxidant activity in pomegranate [42]. The presence of antioxidant compounds will lead the plant to have a better morphological performance [8], stimulate growth, and enhance tolerance to environmental conditions [43]. These catechin units can function as antioxidants in various organisms [44].

### 3.3. Fermented Extract Mineral Composition

The process of fungal fermentation allowed the biotransformation of pomegranate residues into compounds with antioxidant activity, which will allow the fermentation extract to function as an inducer of resistance in the cultivation of plants. Another important analysis was the mineral composition derived from the fermentation process (Table 2), which is innovative process to improve wastes with poor mineral contents for plant nutrition.

**Table 2:** Mineral analysis, antioxidant activity, condensed tannins, and hydrolyzable tannins of the fermented pomegranate extract at 0 and 96 h.

Determination	Units	0 h	96 h
Cond. Electric	dS m^−1^	0.87 ± 0.0435^b^	1.3 ± 0.065^a^
Nitrogen (N)	%	0.01 ± 0.0005^a^	0.01 ± 0.0005^a^
Potassium (K)	%	0.01 ± 0.0005^a^	0.01 ± 0.0005^a^
Phosphorus (P)	%	0.005 ± 0.00025^b^	0.0061 ± 0.00031^a^
Magnesium (Mg)	%	0.0004 ± 0.00002^b^	0.0006 ± 0.00003^a^
Sulfur (S)	%	0.0008 ± 0.00004^b^	0.0011 ± 0.00006^a^
Iron (Fe)	ppm	0.92 ± 0.046^a^	0.71 ± 0.0355^b^
Manganese (Mn)	ppm	0.24 ± 0.012^a^	0.25 ± 0.0125^a^
Zinc (Zn)	ppm	0.03 ± 0.0015^b^	0.05 ± 0.0025^a^
Antioxidant activity	mM TE gds^−1^	27.93 ± 5.93^a^	6.47 ± 3.55^b^
Condensed tannins	mg CE gds^−1^	10.56 ± 0.36^b^	30.43 ± 3.83^a^
Hydrolyzable tannins	mg GAE gds^−1^	141.35 ± 8.15^a^	160.51 ± 21.15^a^

*Note:* Means that do not share a letter are significantly different.

Comparing an aqueous extract of the residue of PGW, against the extract of the fermentation process, it is possible to enhance the mineral content with the fermentation process. The main characteristics were improved where the electric conductivity is 49%, the phosphorus, magnesium, sulfur, and zinc are 22%, 50%, 38%, and 67%, respectively. The HPP hydrolysis is derived in a 38% increase in antioxidant capacity. Iron is an important mineral for crops, which had a decrease in its content of −23%, possibly due to chelation with polyphenol derivatives, since these have a great iron chelating capacity [45]. The largest compounds that were increased in the extract obtained at 96 h of the fermentation process were the condensed tannins (162%), measured as catechin units.

### 3.4. Agronomic Variables

**Figure 5:**
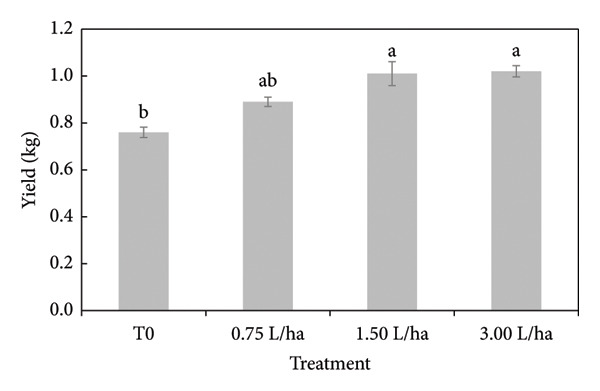
Yield of tomato cultivation applied with different concentrations of the fermented extract of PGW. Means that do not share a letter are significantly different.

For the variable root dry weight, no significant differences were found between the treatments, while for the rest of the variables, at least one treatment was different (Table 3). In the variable plant height, it was observed that when applying the treatment of 3.00 L ha^−1^ of the FPE, an increase of 8% was obtained with respect to the absolute control (T0). Regarding the number of leaves on the plant, treatments with FPE obtained 6% more leaves compared to T0. On the other hand, the variable fresh aerial biomass and dry aerial biomass presented significant differences when comparing the application of FPE against the absolute control. The treatment of 0.75 L ha^−1^ slightly increased 1% by weight for both variables. For the fresh root weight variable, the treatment of 0.75 L ha^−1^ of FPE increased by 13% of dry weight compared to T0. The crop yield variable (Figure 5) was observed as the application of the FPE increased; an increment of up to 34% in yield was obtained compared to the T0.

**Table 3:** Effect of the application of the fermented extract of pomegranate on the growth, development, and productivity of tomato plants.

Treatments	SD	H	NL	FAB	DAB	FRW	DRW
T0	12.5 ± 0.83^b^	94.3 ± 3.1^b^	15.3 ± 0.6^b^	1.31 ± 0.13^a^	181.3 ± 19^a^	74.1 ± 26^ab^	28.6 ± 10.8^a^
0.75 L ha^−1^	13.6 ± 0.69^a^	100.1 ± 5.0^a^	16.2 ± 0.7^a^	1.32 ± 0.14^a^	182.6 ± 19^a^	83.6 ± 17^a^	28.7 ± 6.0^a^
1.50 L ha^−1^	13.9 ± 0.65^a^	99.1 ± 4.3^a^	16.2 ± 0.7^a^	1.12 ± 0.25^b^	147.3 ± 30^b^	62.1 ± 22^b^	23.4 ± 8.1^a^
3.00 L ha^−1^	13.6 ± 0.76^a^	101.7 ± 3.0^a^	16.1 ± 0.4^a^	1.22 ± 0.17^ab^	168.1 ± 21^ab^	83.8 ± 23^a^	29.4 ± 8.5^a^

*Note:* Means that do not share a letter are significantly different. SD: stem diameter (mm); height (cm); NL: number of leaves; FAB: fresh aerial biomass (kg); DAB: dry aerial biomass (g); FRW: fresh root weight (g); DRW: root dry weight (g). Means with different letters within the same column are significantly different (*p* ≤ 0.05).

Pomegranate residues have been shown to be rich in phenolic compounds, mainly gallic acid, catechin, punicalagin, cyanidin‐3‐o‐glu, and quercetin [46]. These secondary metabolites induce the proper functioning of plants and can be used as biostimulants due to the fact that they exert several positive effects on the plant [47, 48], such as having a high antioxidant capacity [49], which helps to protect the various organs of the plant from oxidation, in addition to promoting lignification, which improves protection against different pathogens by strengthening the barrier of the plant cell wall [50]. Villanueva‐Couoh et al. [51] observed that when applying phenolic compounds foliar to chrysanthemum plants, higher plant height, stem diameter, and higher biomass production are obtained; these results are like those obtained in this investigation. Similarly, do Nascimento Silva et al. [52] reported that the foliar application of phenolic compounds improved the fresh and dry matter of the sprout, increased the volume and area of the root, favored less perspiration, and improved the length of bean plant pods (*Phaseolus vulgaris* L.).

### 3.5. Fruit Quality

**Figure 6:**
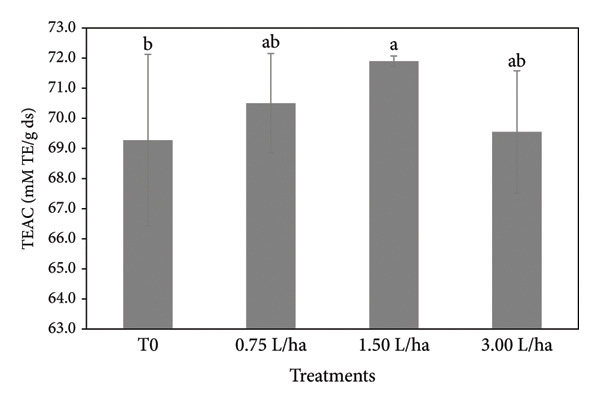
Antioxidant capacity activity of tomato treated with fermented broth at different concentration conditions. Means that do not share a letter are significantly different.

For the fruit quality variables, significant differences were obtained between treatments (Table 4). The fruits that showed the greatest firmness where the ones that the FPE were applied, being the treatment of 3.00 L ha^−1^ that obtained an average of 5.35 kg cm^−1^, compared to the T0 that obtained an average of 4.30 kg cm^−1^. For the TSS variable, by applying a higher concentration of FPE, an increase of 7% compared to T0 was obtained. On the other hand, when applying the FPE, a slight decrease of 1%–2% is obtained for the variables of pH and titratable acidity. In the lycopene (Table 4) and antioxidant capacity (Figure 6) variables, the application of the FPE leads to an increase by 17% and 1%, respectively, compared against T0.

**Table 4:** Fruit quality of tomato plants treated with fermented pomegranate extract.

Treatments	F	SS	pH	TA	Lycopene	RSC
T0	4.30 ± 0.88^c^	5.00 ± 0.28^b^	4.36 ± 0.037^a^	0.30 ± 0.036^a^	1.33 ± 0.32^b^	90.89 ± 0.91^b^
0.75 L/ha	5.97 ± 1.15^ab^	5.28 ± 0.22^ab^	4.32 ± 0.040^ab^	0.27 ± 0.030^ab^	1.19 ± 0.30^b^	91.28 ± 0.53^ab^
1.50 L/ha	6.05 ± 2.14^a^	5.27 ± 0.30^ab^	4.30 ± 0.038^b^	0.28 ± 0.038^ab^	1.26 ± 0.29^b^	91.73 ± 0.06^a^
3.00 L/ha	4.50 ± 1.04^bc^	5.35 ± 0.34^a^	4.30 ± 0.036^b^	0.25 ± 0.030^b^	1.76 ± 0.54^a^	90.98 ± 0.65^ab^

*Note:* F: firmness (kg/cm); SS: soluble solids (°Brix); TA: titratable acidity (citric acid percent). Lycopene is expressed as mg/g ds; RSC: radical scavenging capacity (%). Means with different letters within same column are significantly different (*p* ≤ 0.05).

Fruit extracts rich in phenolic compounds can also increase the quality and firmness of the fruit as they help to maintain the integrity of the membrane and improve the ability to remove reactive oxygen species (ROS) [53]. The increase in TSS due to the application of extracts is due to the fact that the FPE improves the metabolic activities that lead to the synthesis of organic acids, metabolites, and glucose, which are the main compounds of TSS [54].

**Figure 7:**
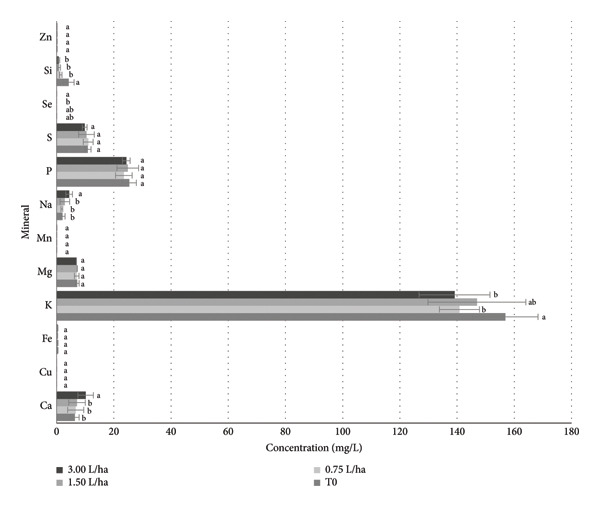
Mineral composition in tomato fruit at different concentrations applied to the fermented PGW extract. Means that do not share a letter are significantly different.

The results of the mineral content in fruit, in copper (Cu), iron (Fe), magnesium (Mg), manganese (Mn), phosphorus (P), sulfur (S), and zinc (Zn) did not show significant differences (Figure 7). For the treatment of 3.00 L ha^−1^ of the FPE, we can observe that the calcium (Ca) increased by 59% compared to the control. On the other hand, potassium (K) decreased from 11% to 6% with the application of FPE. The sodium (Na) presented an increase of 112% with the treatment of 3.00 L ha^−1^ of the FPE. For selenium mineral (Se), an increase of 14% was obtained compared to the control, and for silicon (Si), it decreased from 100% to 18% of the content of this mineral when the FPE was applied.

Increases in the content of calcium, sodium, and selenium are favorable in human nutrition since they take part in important metabolic functions. Calcium aids in muscle function, nervous stimulation, enzymatic and hormonal activities, and oxygen transport. A selenium deficiency, although it is a trace mineral, can cause heart disease and is associated with several types of cancers [55].

When applying different concentrations of FPE, we can see that some of the evaluated variables are favored since they contain compounds that can serve as antioxidants due to their ability to inhibit the formation of radical species, inhibit enzymes related to the production of reactive species of oxygen and superoxide anions, by chelation of metal ions such as iron and copper [56]. This implies that the phenolic compounds present in the crude extract perform essential functions in the cell wall, such as providing both physical and chemical barriers, protection against pathogen invasion, and astringency that prevents attack by insects and animals [57].

## 4. Conclusions

The liquid fermentation process of PGW residues by the fungus *Aspergillus niger* was able of lead to an extract rich in phenolic compounds with antioxidant capacity. During the bioprocess, it was possible to modify the mineral composition of the pomegranate residues. The foliar application of a fermented extract of pomegranate residues, which contains phenolic compounds and antioxidant capacity, was able to increase the yield of the tomato crop and the nutritional quality of the tomato fruits. The fermentative process using agro‐industrial waste is an alternative for the generation of agricultural inputs through sustainable processes, giving added value to these wastes.

## Conflicts of Interest

The authors declare no conflicts of interest.

## Author Contributions

Gilberto Abdón‐Aguilar: methodology, investigation, data acquisition, validation, and writing–original draft; Ana L. Rueda‐Altunar: methodology, investigation, and data acquisition; Susana González‐Morales: visualization, supervision, resources, and project administration; Antonio Juárez‐Maldonado: resources and writing–review and editing; Adalberto Benavides‐Mendoza: writing–review and editing. Marcelino Cabrera‐De la Fuente: supervision and writing–review and editing; Ana Verónica Charles‐Rodríguez: validation and writing–review and editing. Armando Robledo‐Olivo: visualization, supervision, data interpretation, funding acquisition, resources, and project administration.

## Funding

This study was funded by the Universidad Autónoma Agraria Antonio Narro, through the Research Directorate, under the project key 38111‐425204001‐2235.

## Data Availability

The authors have nothing to report.
